# Association Between the Immune Checkpoint Inhibitor Durvalumab and Myasthenia Gravis: A Comprehensive Review

**DOI:** 10.7759/cureus.68542

**Published:** 2024-09-03

**Authors:** Gaurav Bector, Shubam Trehan, Mahyar Toofantabrizi, Gurjot Singh, Aayush Jain, Nirav Arora, Suchitra Shrestha, Gianeshwaree Alias rachna Panjwani, Prateek Jain, Eva Kalra

**Affiliations:** 1 Medicine and Surgery, Dayanand Medical College and Hospital, Ludhiana, IND; 2 Internal Medicine, Dayanand Medical College and Hospital, Ludhiana, IND; 3 Internal Medicine, Union Memorial Hospital, Medstar Health Baltimore, Baltimore, USA; 4 Internal Medicine, Adesh Institute of Medical Science and Research, Bathinda, IND; 5 Computer Science, Lamar University, Beaumont, USA; 6 Internal Medicine, Nepalese Army Institute of Health Sciences, Kathmandu, NPL; 7 Medicine, Ghulam Muhammad Mahar Medical College, Sukkur, PAK; 8 Internal Medicine, All India Institute of Medical Sciences, Rishikesh, IND

**Keywords:** immune-related adverse events (iraes), immune checkpoint inhibitor, medication side effect, adverse side effect, durvalumab, myasthenia gravis

## Abstract

Immune checkpoint inhibitors (ICIs), including Imfinzi (durvalumab), have revolutionized cancer treatment by stimulating the body's immune system to target cancerous cells. Although pharmaceuticals offer therapeutic benefits, several drugs have been associated with immune-related adverse events (irAEs), including the uncommon but serious condition known as myasthenia gravis (MG). This review synthesizes data from pertinent research to offer a thorough evaluation of the literature on the underlying mechanisms, clinical manifestations, and therapeutic approaches for durvalumab-induced MG. The incidence of MG in patients on durvalumab and other ICIs is typically low, with less than 1% documented, despite the potential for severe problems associated with the disease. Durvalumab disrupts immunological tolerance by stimulating autoreactive T-cells and inducing the production of autoantibodies. The clinical consequences of MG need meticulous monitoring, prompt identification, and suitable management to efficiently control the condition. Medical practitioners must carefully weigh the positive effects of ICIs against the possible hazards, emphasizing the necessity for more extensive investigation to improve patient results and establish uniform treatment protocols.

## Introduction and background

Durvalumab is a monoclonal antibody that acts as an immune checkpoint inhibitor (ICI), a type of medication that has significantly transformed cancer therapy by stimulating the body's immune system to attack cancerous cells. Immune checkpoint inhibitors, such as durvalumab, have demonstrated significant potential in treating different types of malignancies by blocking inhibitory mechanisms that restrict the activation of T-cells, hence boosting the immune response against cancerous cells [1]. Traditionally, cancer treatment mostly relied on surgical procedures, radiation therapy, and chemotherapy, which frequently exhibited restricted effectiveness and significant adverse reactions [2-3]. The discovery of ICIs brought about a significant change in the field, as many patients experienced extended periods of remission and continued positive responses [4-5]. The objective of this study is to comprehensively assess the correlation between Imfinzi (durvalumab) and myasthenia gravis (MG), an uncommon yet serious immune-related adverse event (irAE) associated with ICIs. Gaining a comprehensive understanding of this connection is crucial for enhancing patient results and providing guidance for therapeutic interventions.

The mechanism of action of ICIs is centered around the ability of malignant cells to evade the immune system. Tumor cells commonly utilize immunological checkpoints as a means to evade identification and eradication by the immune system. Durvalumab hinders the evasion of the immune system by obstructing the interaction between programmed cell death ligand-1 (PD-L1) and programmed cell death protein-1 (PD-1), therefore enabling T-cells to identify and eliminate cancer cells [6]. Imfinzi has played a vital role in the treatment of non-small cell lung cancer (NSCLC). It is specifically recommended for patients with locally advanced, unresectable illnesses who have not had progression after receiving platinum-based chemoradiation therapy [7]. Although the therapeutic advantages of ICIs are well acknowledged, their utilization is not devoid of hazards. An important issue to consider is the rise of irAEs, which result from heightened immunological activity. Myasthenia gravis has been identified as a significant yet atypical adverse effect [8]. Myasthenia gravis is an autoimmune disorder characterized by the weakening and tiredness of skeletal muscles. This occurs when autoantibodies impair or obstruct acetylcholine receptors at the junction between nerves and muscles (neuromuscular junction). Myasthenia gravis is a result of an abnormal immune response where the immune system mistakenly recognizes parts of the neuromuscular junction, leading to a decrease in the transmission of signals from nerves to muscles. From a clinical perspective, this presents as different degrees of muscular weakness and exhaustion, which can range from visual symptoms like drooping eyelids and double vision to more severe impairment of the respiratory muscles [9]. The specific mechanisms through which ICIs like durvalumab cause MG are currently under investigation. However, it is anticipated that increased activation of T-cells will play a role in disrupting immunological tolerance, leading to autoimmune reactions against components of the neuromuscular junction [10].

Gaining a comprehensive understanding of the detrimental impacts of ICIs is of utmost importance for several compelling reasons. For instance, it affects clinical practice by emphasizing the importance of continuous monitoring and prompt intervention in patients who are prescribed these medications. Furthermore, it provides insights into the wider consequences of immune system modulation in cancer therapy, hence aiding the advancement of safer and more efficient treatment procedures [11]. Although there is a substantial amount of research on the effectiveness and safety of ICIs, there are still notable deficiencies in our comprehension, specifically on the mechanisms responsible for irAEs and the most effective approaches for managing them [12]. Prior research has demonstrated instances of MG linked to ICIs, specifically durvalumab. Makarious et al. [13] documented the frequency of MG in patients using PD-1 and PD-L1 inhibitors, emphasizing the need for healthcare professionals to be knowledgeable about and apply therapeutic approaches. Nevertheless, these studies often encounter constraints, such as limited sample numbers, retrospective designs, and variations in diagnostic criteria and therapeutic regimens, all of which might impact the applicability and dependability of their results [14].

The advent of ICIs has profoundly revolutionized the field of cancer treatment. These drugs, especially durvalumab, have broadened the therapy options for formerly challenging-to-control malignancies like NSCLC. The approval of durvalumab for the treatment of patients with locally advanced, unresectable NSCLC who have not seen progression after platinum-based chemoradiation therapy is a significant achievement in the field of oncology [7]. Nevertheless, the probability of irAEs, especially in autoimmune disorders like MG, requires a careful evaluation of the benefits and risks associated with these therapies. The occurrence of MG in individuals treated with ICIs exemplifies the complexities of immune system control. This infrequent occurrence, albeit uncommon, elicits significant apprehension because of its potential gravity and the intricacy of its management. Healthcare professionals should possess the ability to promptly recognize initial indications of MG and employ suitable interventions to mitigate its effects on patients' quality of life [15]. This review will provide an in-depth analysis of the underlying physiological mechanisms, observable symptoms, and treatment approaches for MG that are linked to the use of durvalumab. It aims to be a valuable reference for both medical practitioners and researchers. The objective is to close information disparities and offer practical perspectives on the secure and efficient utilization of ICIs, consequently aiding in the continuous endeavors to enhance cancer treatment.

## Review

Molecular mechanisms/ pathophysiology

Durvalumab pathophysiology and its relationship with MG may be understood by delving into the molecular processes and immunological pathways involved. Understanding these pathways is essential for creating effective management plans and improving patient outcomes.

Mechanism of Action of Durvalumab

Imfinzi is a kind of human monoclonal antibody that exclusively focuses on PD-L1, which is an immunological checkpoint protein that is located on the surfaces of many cells, such as tumor cells and antigen-presenting cells (16). In typical physiological conditions, PD-L1 binds to its receptors, PD-1 and CD80, on T-cells. This link transmits inhibitory signals that reduce the rate of T-cell multiplication, cytokine generation, and cytotoxic activity. This helps maintain a balanced immune system and prevents the occurrence of autoimmune diseases [16]. Durvalumab exhibits a strong binding affinity to PD-L1, effectively preventing its interactions with PD-1 and CD80. Durvalumab blocks this pathway, so inhibiting the inhibitory signals mediated by PD-L1, resulting in enhanced activation of T-cells and promoting a more robust immune response against tumors (Table 1) [17]. 

**Table 1:** Mechanisms of action of immune checkpoint inhibitors ICI: immune checkpoint inhibitor; PD-L1: programmed death-ligand 1; PD-1: programmed death-1; CD80 cluster of differentiation 80; PD-L2: programmed death-ligand 2; CTLA-4: cytotoxic T-lymphocyte-associated protein 4; B7: B7 molecules; CD80/CD86; APC: antigen-presenting cells

ICI	Target	Mechanism of action	Impact on immune modulation
Imfinzi	PD-L1	Binds to PD-L1, blocking its interaction with PD-1 and CD80	Enhances T-cell activation and proliferation, reduces T-cell exhaustion, and promotes anti-tumor response
Pembrolizumab	PD-1	Binds to PD-1 receptor on T-cells, blocking interaction with PD-L1 and PD-L2	Prevents T-cell inhibition, enhances T-cell function, promotes immune response against tumors
Nivolumab	PD-1	Binds to PD-1 receptor on T-cells, blocking interaction with PD-L1 and PD-L2	Restores T-cell activity, reduces immune suppression, and promotes recognition and destruction of cancer cells
Ipilimumab	CTLA-4	Binds to CTLA-4 on T-cells, blocking interaction with B7 molecules on antigen-presenting cells	Inhibits regulatory T-cell function, enhances effector T-cell activity, and amplifies immune response against tumors

Immune Checkpoint Inhibition and Immune-Mediated Adverse Reactions

While blocking immunological checkpoints like PD-1/PD-L1 might enhance the body's ability to fight against tumors, it can also disrupt the immune system's ability to tolerate normal tissues, leading to irAEs [18]. These adverse consequences occur because the same systems that regulate immune responses to cancerous growths also have a significant function in preventing the occurrence of autoimmune diseases. If these checkpoints are disturbed, the immune system may exhibit an exaggerated response, attacking not just cancer cells but also healthy tissues and organs [19]. Checkpoint inhibitors' heightened T-cell activation can lead to a range of irAEs, such as skin, gastrointestinal, liver, endocrine gland, and neuromuscular junction complications. The unfavorable results often arise from the activation of autoreactive T-cells and the production of autoantibodies, which specifically attack self-antigens and cause harm to tissues [20].

Specific Pathophysiological Mechanisms Linking Durvalumab to Myasthenia Gravis

Myasthenia gravis is an autoimmune disease characterized by the production of autoantibodies against acetylcholine receptors (AChRs) or other components of the neuromuscular junction, such as muscle-specific kinase (MuSK). These autoantibodies disrupt the process of transmitting signals between nerves and muscles, resulting in muscle weakness and fatigue [21]. The connection between durvalumab and myasthenia gravis is established through many molecular and immunological pathways. Durvalumab hinders the PD-1/PD-L1 pathway, which disrupts the processes that maintain immunological tolerance and prevent the occurrence of autoimmunity. The PD-1 signaling pathway plays a crucial role in maintaining the equilibrium between immune activation and tolerance. Inhibiting this route can trigger autoreactive T lymphocytes, which specifically attack components of the neuromuscular junction. As a result, durvalumab enhances the activation, growth, and production of cytokines in T-cells. Some individuals may have increased immune activity that might trigger autoreactive T-cells, which identify AChRs or MuSK as antigens. Autoreactive T-cells have the ability to initiate and spread an autoimmune response, leading to the production of harmful autoantibodies. Durvalumab's augmentation of T-cell function can also stimulate B cells to produce autoantibodies targeting AChRs or MuSK. The autoantibodies bind to their targets in the neuromuscular junction, preventing acetylcholine from binding, forming connections between receptors, and leading to their internalization and death. This process results in reduced neuromuscular transmission and the manifestation of clinical symptoms associated with myasthenia gravis. In addition, the inhibition of PD-1/PD-L1 might augment the synthesis of pro-inflammatory cytokines, such as interferon‐gamma (IFN‐γ) and tumor necrosis factor-alpha (TNF-α). These cytokines can worsen inflammation and tissue damage at the neuromuscular junction, hence further contributing to the advancement of myasthenia gravis.

Multiple investigations have elucidated the molecular and immunological mechanisms linking durvalumab to MG. Haratani et al. [22] discovered occurrences of MG in patients who received PD-1/PD-L1 inhibitors, highlighting the importance of immunological tolerance mechanisms in preventing autoimmunity. Similarly, Makarious et al. [13] highlighted the increasing toxicity of MG when ICIs are used. They underlined the need for early identification and management in clinical practice. Dalakas [23] conducted research on the immunopathogenesis of MG induced by ICIs. The study emphasized that these drugs can lead to heightened activation of T-cells and increased production of cytokines. As a consequence, immunological tolerance is compromised and autoreactive immune responses are triggered. Vincent et al. [24] examined the role of autoantibodies in the progression of MG, providing a theoretical basis for comprehending how durvalumab-induced immune activation may contribute to the development of this autoimmune disease.

The occurrence of MG as a result of durvalumab is characterized by a complex interplay of molecular and immunological mechanisms. Durvalumab inhibits the PD-1/PD-L1 pathway, leading to enhanced activation of T-cells. This can disrupt immunological tolerance, trigger the activation of self-reactive T-cells, the production of harmful autoantibodies, and inflammation mediated by cytokines. These pathways collectively lead to the development of MG in specific individuals who are taking durvalumab, underscoring the significance of constantly monitoring and treating this adverse event.

Discussion

The existing body of evidence about the correlation between durvalumab and MG demonstrates an intricate interaction of immunological systems. The literature reveals a diverse range of findings, highlighting the complexity of irAEs associated with ICIs. The evaluated trials consistently demonstrate that durvalumab effectively enhances the body's immune response against tumors. However, it also carries the potential danger of causing irAEs, such as MG. For instance, the PACIFIC trial conducted by Antonia et al. (2018) reported that the incidence of MG in patients treated with durvalumab was less than 1%, a finding that underscores the rarity but significant clinical implications of this condition. The prevalence of MG in individuals who received durvalumab and other ICIs is typically low, with reports of fewer than 1% of patients in clinical studies [7]. Nevertheless, the seriousness of myasthenia gravis when it arises requires a cautious and aggressive approach to its therapy.

Comparison of Studies’ Methodologies, Results, and Conclusions

The studies employ many approaches, including clinical trials, retrospective analyses, case reports, and reviews. The variety of study designs contributes to the wide range of data available, but it also provides unpredictability that can make direct comparisons more complicated. For example, while the PACIFIC study employed a randomized controlled trial design that provided robust data on the efficacy and safety of durvalumab, other studies, such as those by Makarious et al. (2017) and Haratani et al. (2018), utilized retrospective analyses and case reports that, while informative, may lack the rigor and generalizability of larger clinical trials. The primary goal of big clinical studies, such as the one done by Antonia et al. [4], was to assess the effectiveness of durvalumab in the treatment of NSCLC. These trials usually involved thorough monitoring to identify any negative effects, resulting in strong evidence of the occurrence of MG and other irAEs. The PACIFIC study, which assessed the use of durvalumab in patients with NSCLC, found that around 15% of patients experienced irAEs. Among these events, MG was a rare but recognized occurrence. Retrospective analyses and case reports provide an in-depth understanding of individual patient experiences and particular adverse occurrences. Haratani et al. [22] performed a retrospective investigation on patients who received ICIs and discovered that the presence of MG was linked to considerable morbidity. This study, along with others like it, highlights the importance of early detection and intervention to prevent severe outcomes. However, the retrospective nature of these studies means they are often limited by biases and lack of control over confounding variables, which can affect the reliability of their conclusions. Case studies, such as the ones published by Makarious et al. [13], offer useful information on the clinical manifestation, development, and treatment of MG in the context of ICI therapy. These findings emphasize the diversity in patient reactions and the necessity for tailored approaches to care [13].

Moreover, comprehensive reviews and meta-analyses, such as those by Dalakas (2019), provide a synthesis of existing research, offering insights into the immunopathogenesis of ICI-induced MG. These works are critical in building a theoretical framework that explains how ICIs might disrupt immune tolerance and trigger autoimmune responses. However, these reviews also point out the gaps in our understanding, particularly regarding the long-term outcomes and management of MG in patients undergoing ICI therapy. Reviews and meta-analyses compile data from several studies to provide thorough summaries of the occurrence, causes, and treatment of irAEs. Dalakas [23] conducted a comprehensive analysis of the immunopathogenesis of ICI-induced MG, with a particular focus on the involvement of autoreactive T-cells and inflammation driven by cytokines. These evaluations provide a broader perspective on individual study findings in the field of ICI research [23]. Regardless of the variations in the way the studies were conducted, a consistent pattern emerges: durvalumab, by its ability to boost T-cell activation and modulate the immune system, might disturb normal immunological tolerance, resulting in autoimmune reactions like MG. This pattern is evident across different study designs and patient populations, reinforcing the notion that while MG is rare, its occurrence is clinically significant and warrants careful monitoring. This highlights the importance of closely observing and promptly intervening in patients who are being administered ICIs.

A summary of key studies on durvalumab and myasthenia gravis is presented in Table 2.

**Table 2:** Summary of key studies on durvalumab and myasthenia gravis NSCLC: non-small cell lung cancer; MG: myasthenia gravis; PD-1: programmed death-1; PD-L1: programmed death-ligand 1; ICI: immune checkpoint inhibitor

Study	Methodology	Patient population	Findings	Conclusions
Antonia et al. (2018) [3]	Randomized controlled trial	NSCLC patients post-chemoradiotherapy	Immune-related adverse events in 15% of patients, MG rare	Durvalumab was effective in NSCLC; MG is a rare but significant adverse event
Michot et al. (2016) [9]	Retrospective analysis	Patients receiving PD-1/PD-L1 inhibitors	MG observed in 0.1-0.2% of patients, associated with significant morbidity	Highlighted the need for early detection and management of MG in patients treated with ICIs
Makarious et al. (2017) [4]	Case reports	Patients with MG induced by ICIs	Detailed clinical manifestations and treatment responses	Emphasized the importance of recognizing MG symptoms early and managing them promptly in ICI-treated patients
Dalakas (2019) [5]	Review	Literature on ICI-induced MG	Mechanisms involving T-cell activation and cytokine production	Provided a comprehensive understanding of the immunopathogenesis of ICI-induced MG, suggesting areas for further research
Vincent et al. (2001) [12]	Review	Patients with autoimmune MG	Role of autoantibodies in MG pathogenesis	Suggested that autoantibody production could be a mechanism in ICI-induced MG, necessitating further investigation

Clinical Implications of the Association between Imfinzi and Myasthenia Gravis

The association between Imfinzi and myasthenia gravis has noteworthy clinical ramifications, especially for patient care and safety. The rarity of MG in the context of ICI therapy, coupled with its potential severity, underscores the need for heightened vigilance among healthcare providers. Due to the potentially serious outcomes of MG, it is essential for healthcare practitioners to have the necessary skills and knowledge to quickly identify and manage this adverse event. Timely identification of MG is essential in order to avert serious problems. It is important to closely observe patients who are being treated with durvalumab for any indications of MG, including muscular weakness, drooping eyelids (ptosis), and difficulty swallowing (dysphagia). Clinical guidelines emphasize the importance of regular neurological assessments and patient education to facilitate early detection. Performing regular neurological examinations and collecting patient-reported outcomes can aid in the early detection of neuromuscular dysfunction [9]. The treatment of ICI-induced MG entails a comprehensive strategy that often includes immunosuppressive medicine and supportive care. High-dose corticosteroids, such as prednisone, are frequently employed to diminish inflammation and the development of autoantibodies. In more severe instances, the use of intravenous immunoglobulin (IVIG) or plasmapheresis may be required to promptly reduce the levels of autoantibodies [10]. However, the balance between managing MG and continuing cancer therapy remains a delicate challenge, and decisions should be made on a case-by-case basis, considering the patient’s overall health and cancer prognosis. Temporary cessation of ICI treatment is frequently necessary until symptoms are managed.

It is crucial to provide patients with information on the possible hazards of ICIs, including MG. Comprehensive informed consent should encompass a thorough conversation on the indications and manifestations of MG, the need for promptly reporting any symptoms, and the probable requirement for immunosuppressive therapies. This enables people to actively engage in their healthcare and instantly communicate any symptoms they have. The difficulty in utilizing ICIs such as durvalumab resides in effectively managing their ability to combat tumors while also considering the potential for irAEs. This challenge highlights the importance of a multidisciplinary approach involving oncologists, neurologists, and immunologists to optimize patient outcomes. Medical professionals must carefully consider the advantages of enhanced cancer results in comparison to the possibility of serious autoimmune consequences. To achieve this balance, it is necessary to have a sophisticated comprehension of the individual patient's risk factors, cancer features, and general health state [6].

Limitations of Current Research and Areas for Further Investigation

Although the existing research offers useful insights into the correlation between durvalumab and MG, there are numerous limitations present. It is crucial to do more study in order to overcome these constraints and improve our comprehension and control of this negative occurrence. One significant limitation is the relatively small sample sizes and the retrospective nature of many studies, which can introduce biases and limit the generalizability of the findings. Several investigations on ICI-induced MG sometimes utilize limited sample sizes or retrospective methodologies, potentially restricting the applicability of the results. Additionally, comprehensive investigations are required to gather more reliable information on the frequency, variables that contribute to, and results of MG in individuals undergoing ICI treatment [7]. The heterogeneity in diagnostic criteria and therapeutic approaches among research hinders direct comparisons and the establishment of consistent guidelines. To address this, there is a need for standardized diagnostic criteria and treatment protocols that can be universally applied, enhancing the consistency and quality of care for patients. Developing standardized criteria for identifying and treating ICI-induced MG will enhance the uniformity and excellence of healthcare [12]. It is crucial to do more studies to better understand the molecular and immunological pathways that cause ICI-induced MG. Gaining knowledge about the precise pathways and immunological processes implicated can aid in the identification of prognostic biomarkers and the creation of focused therapies to avoid or alleviate this unfavorable occurrence [8]. Longitudinal studies are necessary to evaluate the long-term results and quality of life of individuals who develop myasthenia gravis following ICI medication. The purpose of this research is to assess the influence of MG on the effectiveness of cancer therapy, the overall survival rate, and the outcomes reported by patients. This will help us gain a thorough grasp of the long-term consequences of this negative occurrence [24]. Moreover, investigating the potential genetic predispositions that may increase the risk of MG in patients receiving ICIs could provide insights into personalized treatment strategies.

Controversies and Differing Opinions within the Research Community

The research has not yet agreed on certain aspects of ICI-induced MG, which highlights the intricate and dynamic character of this subject. There is a continuing discussion over the actual occurrence of MG in individuals who receive ICIs and the determination of certain risk factors. Several studies indicate that individuals with pre-existing autoimmune disorders or certain genetic predispositions may have an elevated risk, however, other investigations have not shown definitive connections [10]. This ongoing debate suggests that future research should focus on identifying definitive risk factors through larger, more controlled studies. Additional investigation is required to elucidate these risk variables and construct prognostic models. There are varying viewpoints about the most effective approach to managing ICI-induced MG. The use of high-dose corticosteroids is frequent, but their effect on cancer outcomes is worrisome. Certain specialists recommend early intervention with IVIG or plasmapheresis to reduce the necessity of long-term corticosteroid usage, while others stress the significance of maintaining a balance between immunosuppression and cancer management [15]. The decision-making process in such cases should involve a careful assessment of the risks and benefits, with close monitoring to ensure patient safety. The choice to provide ICIs again to patients who have experienced MG is a subject of debate and disagreement. Certain medical professionals advocate for rechallenge in meticulously chosen individuals with vigilant supervision, asserting that the advantages of cancer management may surpass the potential hazards of recurring MG. Some individuals advise against rechallenge because of the possibility of experiencing serious and life-threatening problems [14].

Statistical Evidence and Implications

Quantitative data obtained from clinical trials and retrospective investigations offer a more precise understanding of the frequency and results of MG linked with ICIs. For instance, the PACIFIC trial provided valuable statistical data indicating that while MG is rare, the overall incidence of irAEs in patients treated with durvalumab was approximately 15%, highlighting the broad spectrum of potential adverse effects. In the PACIFIC study, immune-related side effects, such as MG, were observed in around 15% of patients who were administered durvalumab. Specifically, MG occurred in fewer than 1% of patients (7). Haratani et al. [22] conducted retrospective studies and discovered that MG occurred in around 0.1%-0.2% of patients who received ICIs. This highlights the infrequency but substantial consequences of this adverse event [22]. These statistical findings underscore the importance of continued research to better quantify the risks and develop strategies for minimizing adverse outcomes. A statistical study further demonstrates that patients who get MG as a result of ICIs frequently necessitate severe immunosuppressive treatment and may have extended hospital stays, leading to escalated healthcare expenses. For instance, Makarious et al. [13] found that a considerable number of patients who had MG needed IVIG or plasmapheresis, and some of them had serious consequences that required urgent care [13].

Clinical Guidelines and Best Practices

It is crucial to develop clinical standards and best practices for managing ICI-induced MG in order to enhance patient outcomes. Current data suggest that it is advisable to do a thorough first examination, which should include a neurological evaluation and screening for any pre-existing autoimmune disorders. It is crucial to educate patients on the indications and manifestations of MG, as well as the need to promptly report any symptoms. Patient education should also emphasize the importance of early symptom recognition, which can lead to timely interventions and prevent severe complications. It is crucial to conduct regular neurological examinations both during and after ICI therapy in order to promptly identify initial indications of MG. It is recommended to promptly start large doses of corticosteroids when symptoms first appear and to consider using IVIG or plasmapheresis in severe instances. It is also advised to temporarily stop ICI therapy until symptoms are under control. These are the recommended techniques for managing this condition. Furthermore, a multidisciplinary approach involving oncologists, neurologists, and other specialists is essential for the optimal management of these patients. It is essential to carefully assess the potential dangers and advantages of reintroducing ICIs to patients who have had MG, and closely monitor them if the decision is made to proceed with the reintroduction.

Future Directions

Future research should focus on several key areas to enhance the understanding and management of ICI-induced MG. Large, prospective cohort studies are needed to better characterize the incidence, risk factors, and outcomes of MG in patients treated with ICIs. Such studies should aim to provide more definitive data that can inform clinical guidelines. Conducting large, prospective cohort studies to better characterize the incidence, risk factors, and outcomes of MG in patients treated with ICIs is essential. Additionally, the identification of biomarkers that can predict the risk of MG and other irAEs is crucial. This could lead to more personalized treatment approaches, where patients at higher risk are closely monitored or provided with preventative strategies. Identifying biomarkers that can predict the risk of MG and other irAEs, enabling personalized treatment approaches, is another important area. Investigating the molecular and immunological mechanisms underlying ICI-induced MG to develop targeted preventive and therapeutic strategies is critical. Assessing the long-term impact of MG on cancer treatment efficacy, overall survival, and quality of life to provide a comprehensive understanding of this adverse event is needed. Developing consensus guidelines for the diagnosis and management of ICI-induced MG to standardize care and improve patient outcomes is also important.

## Conclusions

The association between durvalumab and MG presents a significant challenge in cancer immunotherapy, necessitating vigilant monitoring and proactive management due to the potential severity of this adverse event. This review has summarized key findings from existing research, highlighting the underlying physiological processes, clinical symptoms, and treatment strategies for MG induced by ICIs. It underscores the critical need to balance the therapeutic benefits of ICIs with the risk of irAEs. Additional research is needed to address current limitations, establish predictive biomarkers, and improve management techniques. Enhanced management strategies can substantially impact patient outcomes by reducing the severity and frequency of adverse events, thus improving the quality of life for patients undergoing cancer immunotherapy. Effective monitoring and timely intervention can prevent severe complications, lower healthcare costs, and enhance patient well-being. Healthcare providers can tailor treatment plans by identifying predictive biomarkers and developing targeted therapies to minimize risks while maximizing therapeutic benefits. This review also calls for ongoing efforts from healthcare professionals and researchers to stay informed about the latest findings and guidelines, investigate the mechanisms of ICI-induced myasthenia gravis, and refine treatment protocols. In summary, ICIs have revolutionized cancer treatment; their use requires careful management to ensure safety and efficacy. The insights from this review can guide clinical practice and future research, ultimately improving patient outcomes and quality of life in cancer therapy.
